# Modulating native GABA_A_ receptors in medulloblastoma with positive allosteric benzodiazepine-derivatives induces cell death

**DOI:** 10.1007/s11060-019-03115-0

**Published:** 2019-02-06

**Authors:** Laura Kallay, Havva Keskin, Alexandra Ross, Manali Rupji, Olivia A. Moody, Xin Wang, Guanguan Li, Taukir Ahmed, Farjana Rashid, Michael Rajesh Stephen, Kirsten A. Cottrill, T. Austin Nuckols, Maxwell Xu, Deborah E. Martinson, Frank Tranghese, Yanxin Pei, James M. Cook, Jeanne Kowalski, Michael D. Taylor, Andrew Jenkins, Daniel A. Pomeranz Krummel, Soma Sengupta

**Affiliations:** 1 Department of Neurology, Emory University School of Medicine, Atlanta, GA USA; 2 Winship Cancer Institute, Emory University School of Medicine, Atlanta, GA USA; 3 Department of Cell Biology, Emory University School of Medicine, Atlanta, GA USA; 4 The Arthur and Sonia Labatt Brain Tumour Research Centre, The Hospital for Sick Children, Toronto, Canada; 5 Developmental & Stem Cell Biology Program, The Hospital for Sick Children, Toronto, Canada; 6 Department of Chemistry and Biochemistry, University of Wisconsin-Milwaukee, Milwaukee, WI USA; 7 Molecular and Systems Pharmacology Graduate Training Program, Graduate Division of Biological and Biomedical Sciences, Laney Graduate School, Emory University, Atlanta, GA USA; 8 Department of Biomedical Engineering, Johns Hopkins University, Baltimore, MD USA; 9 Electrical and Computer Engineering Department, Boston University, Boston, MA USA; 10 Center for Cancer and Immunology Research, Brain Tumor Institute, Children’s National Medical Center, Washington, DC USA; 11 Department of Biostatistics & Bioinformatics, Rollins School of Public Health, Emory University, Atlanta, GA USA; 12 Division of Neurosurgery, The Hospital for Sick Children, Toronto, Canada; 13 Departments of Anesthesiology & Pharmacology, Emory University School of Medicine, Atlanta, GA USA; 14 Department of Hematology & Medical Oncology, Emory University School of Medicine, Atlanta, GA USA; 15 Department of Neurosurgery, Emory University School of Medicine, Atlanta, GA USA; 16 Winship Cancer Institute, Emory University Hospital, 1365C Clifton Road, Suite C5086, Atlanta, GA USA

**Keywords:** Benzodiazepine, Medulloblastoma, GABA_A_ receptor, Apoptosis, *TP53*

## Abstract

**Purpose:**

Pediatric brain cancer medulloblastoma (MB) standard-of-care results in numerous comorbidities. MB is comprised of distinct molecular subgroups. Group 3 molecular subgroup patients have the highest relapse rates and after standard-of-care have a 20% survival. Group 3 tumors have high expression of *GABRA5*, which codes for the α5 subunit of the γ-aminobutyric acid type A receptor (GABA_A_R). We are advancing a therapeutic approach for group 3 based on GABA_A_R modulation using benzodiazepine-derivatives.

**Methods:**

We performed analysis of *GABR* and *MYC* expression in MB tumors and used molecular, cell biological, and whole-cell electrophysiology approaches to establish presence of a functional ‘druggable’ GABA_A_R in group 3 cells.

**Results:**

Analysis of expression of 763 MB tumors reveals that group 3 tumors share high subgroup-specific and correlative expression of *GABR* genes, which code for GABA_A_R subunits α5, β3 and γ2 and 3. There are ~ 1000 functional α5-GABA_A_Rs per group 3 patient-derived cell that mediate a basal chloride-anion efflux of 2 × 10^9^ ions/s. Benzodiazepines, designed to prefer α5-GABA_A_R, impair group 3 cell viability by enhancing chloride-anion efflux with subtle changes in their structure having significant impact on potency. A potent, non-toxic benzodiazepine (‘KRM-II-08’) binds to the α5-GABA_A_R (0.8 µM EC_50_) enhancing a chloride-anion efflux that induces mitochondrial membrane depolarization and in response, *TP53* upregulation and p53, constitutively phosphorylated at S392, cytoplasmic localization. This correlates with pro-apoptotic Bcl-2-associated death promoter protein localization.

**Conclusion:**

*GABRA5* expression can serve as a diagnostic biomarker for group 3 tumors, while α5-GABA_A_R is a therapeutic target for benzodiazepine binding, enhancing an ion imbalance that induces apoptosis.

**Electronic supplementary material:**

The online version of this article (10.1007/s11060-019-03115-0) contains supplementary material, which is available to authorized users.

## Introduction

Medulloblastoma is a significant cause of cancer-related morbidity and mortality in children [1]. Its standard-of-care consists of surgical resection, followed by radiotherapy and chemotherapy, which cause neurocognitive side effects [2–4]. Medulloblastoma molecular profiling delineated four subgroups, by consensus termed wingless (WNT), sonic hedgehog (SHH), group 3, and group 4 [5–7]. WNT and SHH exhibit anomalous expression of genes associated with the Wnt and Shh pathways, consistent with genomic alterations [8–10]. Groups 3 and 4, which account for ~ 60% of medulloblastomas and include those with poorest prognosis, do not have shared subgroup-specific genomic alterations [10]. Group 3 is often referenced as *MYC*-driven, however *MYC* expression is seen in only a subset of group 3 tumors [11]. Group 3 tumors are typically *TP53* wild-type and its high expression is associated with poor prognosis [12, 13]. Group 3 tumors share high expression of *GABRA5*, which codes for the α5-subunit of the ligand-gated ionotropic γ-aminobutyric acid type A receptor (GABA_A_R) [6].

**Fig. 1:**
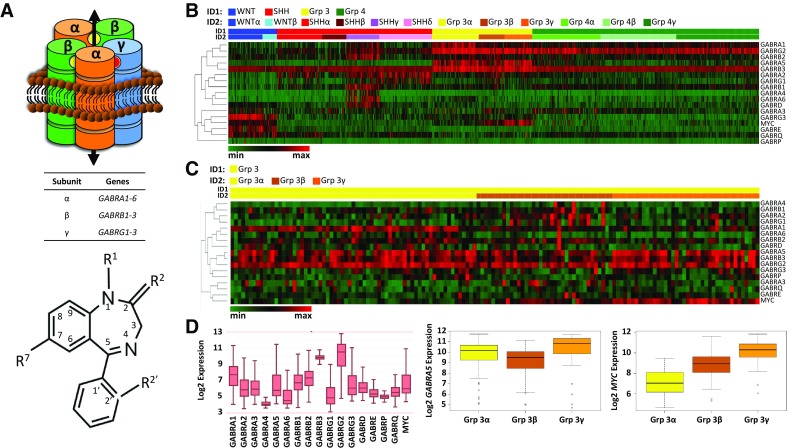
GABA_A_ receptor subunit gene (*GABR*) and *MYC* expression across 763 primary medulloblastoma tumors. **a** Top, GABA_A_ receptor (GABA_A_R), αβαβɣ subunit stoichiometry, consists of five subunit transmembrane segments which create the chloride-anion conduction pore. Inter-subunit binding sites for GABA and benzodiazepine are shown as yellow and red spheres, respectively. Bottom, common core structure of a ‘benzodiazepine’. Indicated are sites frequently modified (R^1^, R^2^, R^2′^, R^7^), which may impart a GABA_A_R subtype-preference. Introduction of an ethinyl bond at R^7^ imparts an α5-GABA_A_R preference. **b** Supervised heatmap clustering analysis across medulloblastoma molecular subgroups using z-score scaling, 1-Pearson correlation distance, and average clustering. The relationship between genes is indicated by the dendrogram (left). Shown bottom, left is a color palette where color scaling indicates low (green) to high (red) expression. Samples were classified into four subgroups (ID1) and further into twelve subtypes (ID2). **c** Supervised heatmap clustering analysis of group 3 only using z-score scaling, 1-Pearson correlation distance, and complete clustering. Shown bottom, left is a color palette where color scaling indicates low (green) to high (red) expression. ID1: group 3, yellow; ID2 within group 3: α, yellow; β, brown; γ, orange. **d** Boxplots of *GABR* and *MYC* expression across subgroups (left) and separately *GABRA5* (middle) and *MYC* (right) expression of group 3

GABA_A_Rs are fundamental in determining an excitation/inhibition balance in the CNS. As an ionotropic receptor mediating chloride-anion flux, GABA_A_Rs predominantly function to hyperpolarize neural cells following binding of γ-aminobutyric acid (GABA), thereby decreasing the likelihood of generating an action potential. GABA_A_R usually consists of two α, two β, and γ subunits arranged as α–β–γ–α–β (Fig. 1a). Nineteen genes encode GABA_A_R subunits, including of six α (*GABRA1-6*), three β (*GABRB1-3*), and three γ (*GABRG1-3*) [14]. Benzodiazepines bind at the γ-α interface and are positive allosteric modulators, acting to increase GABA effectiveness and thus chloride-anion flux. Benzodiazepines consist commonly of fusion of diazepine and benzene rings (1,4-benzodiazepine) and a phenol ring (5-phenyl-1*H*-benzo[*e*]) (Fig. 1a). Changes to its chemical structure can alter its GABA_A_R-subtype preference. For example, introducing an ethinyl bond to the diazepine ring at R^7^ results in a α5-GABA_A_R preference [15, 16].

Investigating GABA_A_R in group 3, we showed that Gabra5 (or α5) was present in patient-derived group 3 cells and tumor tissue and contributed to assembly of a functional GABA_A_R [17]. An α5-GABA_A_R preferring benzodiazepine was capable of impairing group 3 cell viability in vitro [17] and its potency in a mouse model was greater than standard-of-care chemotherapeutic [18] and agents proposed as potential medulloblastoma therapeutics [19, 20]. The most efficacious α5-GABA_A_R preferring benzodiazepine tested (‘QH-II-066’) caused cell cycle arrest and its effectiveness in inducing apoptosis abrogated by loss in expression of HOXA5, a homeobox transcription factor that regulates p53 expression [17]. Further, QH-II-066 sensitized group 3 cells to radiation and cisplatin in a p53-dependent manner. Thus, p53 appears important in group 3 cells’ response to GABA_A_R mediated chloride-anion flux.

We report on analysis of GABA_A_R and *MYC* expression in 763 primary medulloblastoma patient tumors, characterization of GABA_A_R in a patient-derived cell line, identification of chemical features critical to α5-GABA_A_R preferring benzodiazepine potency, and examination of how such benzodiazepines may impair group 3 cell viability.

## Materials and methods

### Gene expression analysis

Normalized gene expression data for sixteen *GABR* genes and *MYC* from 763 primary resected medulloblastoma specimens was used [11]. Samples were classified into four medulloblastoma subgroups and further into twelve subtypes: two WNT subgroup [α (*n* = *49*), β (*n* = *21*)], four SHH subgroup [α (*n* = *65*), β (*n* = *35*), γ (*n* = *47*), δ (*n* = *76*)], three group 3 subgroup [α (*n* = *67*), β (*n* = *37*), γ (*n* = *40*)] and three group 4 subgroups [α (*n* = *98*), β (*n* = *109*), γ (*n* = *119*)]. Heatmaps for analysis of expression across all four subgroups and among group 3 subtypes were generated using Morpheus (https://software.broadinstitute.org/morpheus). Boxplots for expression analysis were created in R.

### Cell lines

Daoy (SHH cell line) and D283 (group 3 cell line) were purchased from ATCC. D425 (group 3 cell line) was obtained through a MTA between Emory and Duke.

### Cell proliferation

Benzodiazepines were synthesized as described [21, 22], kept lyophilized at room temperature, and suspended prior to use in dimethyl sulfoxide (DMSO; 0.125%). D283 cell viability was assayed using the CellTiter 96^®^ AQueous One Solution Assay (Promega) as described [17]. D283 cells (7500) added per well of a Falcon^®^ 96 well flat bottom TC-treated polystyrene cell culture plate (Corning) in pentaplicates and incubated 4–5 h, 37 °C. DMEM (Thermo-Fisher), lacking phenol-red, HEPES, and penicillin/streptomycin but with 20% FBS and 4 mM L-glutamine, was used for plating. Benzodiazepines were diluted in DMSO (0.125%) to a 4 mM working stock for drug dilution in DMEM. After 48 h at 37 °C, 20 µL CellTiter 96^®^ AQueous One Solution (Promega) was added per well, plate incubated 1 h at 37 °C, and absorbance (490 nm) measured. To obtain a reading, media control (average reading of wells containing only media) was subtracted from DMSO control and drug-treated values. Drug-treated values were divided by DMSO values to normalize data. IC_50_ values were obtained using the ‘[Inhibitor] versus normalized response’ nonlinear regression function in Prism 7 software (GraphPad).

### Electrophysiology

Recordings used methods similar to those described [23]. Experiments were performed 24–72 h post-plating at 22 °C and across multiple days (controlling for cell health and expression efficiency). All reagents were purchased from Sigma, unless otherwise noted. Patch pipettes were fabricated from thin-walled borosilicate glass (World Precision Instruments) using a horizontal puller (Sutter Instruments) to give a resistance of 2–8 MΩ when filled with intracellular solution (120 mM KCl/2 mM MgCl_2_/10 mM EGTA/10 mM HEPES, NaOH adjusted to pH 7.2, 315 mOsm). Extracellular solution contained: 161 mM NaCl/3 mM KCl/1 mM MgCl_2_/1.5 mM CaCl_2_/10 mM HEPES/6 mM d-glucose, NaOH adjusted to pH 7.4 (320–330 mOsm). A rapid solution changer (BioLogic Science Instruments) connected to a infusion pump (KD Scientific) delivered GABA and benzodiazepine solutions.

### Mitochondria structure–function

Mitochondrial membrane potential was measured using the TMRE Mitochondrial Membrane Potential Assay Kit (Abcam). D283 cells were treated with drug or control solutions (10 min, 37 °C), 50 nM TMRE added (20 min, 37 °C), and TMRE fluorescence visualized (Leica SP8) and quantified (LAS X platform, Leica).

### Quantitative RT-PCR

Total RNA was extracted from cells (RNeasy Mini Kit, Qiagen), converted into cDNA by PCR (Cloned AMV First-strand Synthesis Kit, Invitrogen; primers shown in Online Table 1), analyzed using SYBR dye (SYBR Green PCR Master Mix, Applied Biosystems).

### Microscopy

Cells plated on poly-d-lysine coated glass coverslips fixed 1 h in 4% (w/v) paraformaldehyde (Electron Microscopy Sciences, EMS), washed in PBS (6×, 5 min/wash), blocked 1 h (PBS, 0.8% Triton X-100, 10% normal goat serum), incubated overnight with antibody. Cells washed in PBS (6×, 5 min/wash) and goat anti-rabbit and goat anti-mouse secondary [Ig-Alexa-488 (green) or Ig-Alexa-555 (red), Invitrogen] added. Cells washed in PBS (6×, 5 min/wash), coverslips mounted on slides (Immuno Mount DAPI and DABCO Mounting Media, EMS), and fluorescence visualized (Leica SP8) and images prepared (LAS X platform, Leica).

### Western blots

D283 whole-cell extracts were prepared as described [17]. Nuclear and cytoplasmic fractions were prepared using NE-PER Nuclear Cytoplasmic Extraction Reagent kit (Thermo-Scientific). Pierce BCA Protein Assay (Thermo-Scientific) was used to quantify protein in lysates. Protein (20 µg whole-cell; 15 µg cytoplasmic/nuclear fractions) were resolved by PAGE using 10% pre-cast gels (Bio-Rad), transferred to 0.45 µM PVDF or 0.45 µM nitrocellulose (for BAD and Caspase-9 antibodies) membranes. Membranes blocked in PBS containing 0.1% Tween 20 and either 5% non-fat dry milk or 5% BSA and probed with primary (anti-p53, anti-PTEN, anti-Caspase-9, anti-GAPDH, anti-β-actin, Lamin B1, PARP, Cell Signaling; anti-MDM2, Abcam). Additionally, Abcam’s p53 Antibody Sampler Panel [S20, S46, S392, phospho-p53 (K382), and p53 (DO)] was used. Membranes washed 3× (10 min/wash) with 0.1% PBST or 0.1% TBST (for phospho-p53 antibodies) and incubated in HRP-conjugated secondary anti-rabbit or anti-mouse (GE). Membranes washed (3×, 10 min/wash) with 0.1% PBST or 0.1% TBST and visualized using ECL Western Blotting Detection Reagent (Amersham) or SuperSignal™ West Pico PLUS Chemiluminescent Substrate (Thermo-Scientific) and film.

## Results

### Medulloblastoma gene expression

We analyzed *GABR* and *MYC* expression across all subgroups in 763 resected primary medulloblastoma tumors [11] (Fig. 1b, c; Online Resource 1, 2; Online Tables 2, 3). This analysis reveals that: (1) all subgroups have shared high expression of select *GABR* genes; (2) there is subgroup-specific high expression of some *GABR* genes and some subgroups have *GABR* expression that is specific to only a subset of patients within the subgroup; (3) there is a positive correlation in expression of *GABRA5* and *MYC* in a subset of group 3 and more surprisingly WNT tumors.

*GABRB3* expression is high across all four subgroups, with subtle differences in the degree of expression across subgroups (Fig. 1b, c). Expression is also high for *GABRG2*, but there is greater variability in degree of expression between subgroups. Groups 3 and 4 have highest expression of *GABRG2*.

*GABR* expression between subgroups and within some subgroups is variable: (i) WNT subgroup subtypes (α and β) have high expression of *GABRG3* and *GABRE*; (ii) SHHγ subtype has high expression of several *GABR* genes that distinguish it from SHHα, SHHβ, SHHδ, while all SHH subgroup patients have high expression of *GABRA2* and *GABRG1*. Medulloblastoma patients with poorest prognosis are group 3. Group 3 patients have high *GABRA5* expression. *GABRA5* expression is consistently the highest in the group 3γ subtype, which carries the poorest prognosis.

Supervised heatmaps and boxplots show expression differences for both *GABRA*5 and *MYC* within group 3 and WNT subgroups. Correlation between *MYC* and *GABRA5* is not statistically significant in group 3 (*p* = 0.202). However, there is a significant positive correlation in expression between *GABRA5* and *MYC* in the group 3α subtype (*p* = 0.006), where it was reported that *MYC* loss is more frequent [9], but not in group 3β (*p* = 0.336). Group 3γ has the highest level of *MYC* expression [11]. We do not find a significant correlation (*p* = 0.634) between *MYC* and *GABRA5* expression in the group 3γ subtype. As well as group 3γ, WNT subgroup patients have high *MYC* expression (Fig. 1b). There is a significant positive correlation of *MYC* and *GABRA5* (*p* < 0.001) in the WNT subgroup (Online Resource 1; Online Table 2), but *GABRA5* expression is significantly lower than in group 3 tumors.

### GABR expression consistent with assembly of α5-GABRAR

To identify the probable composition of a GABA_A_R in medulloblastoma tumors that would be sensitive to benzodiazepine modulation, we examined correlation in expression of GABA_A_R subunits in subgroups using the normalized gene expression dataset of 763 medulloblastoma tumors [11] (Online Resource 2). Using a Spearman’s correlation test (where *p* < 0.01) we find that: (1) there is a positive correlation in all subgroups in expression of *GABR* genes that may form a functional GABA_A_R sensitive to benzodiazepine modulation; and (2) group 3 has a high and correlative expression that includes *GABRA5*. In WNT, SHH, group 3, and group 4 there is a shared correlation in expression of two groups of genes that suggest assembly of a functional GABA_A_R and its composition. The *GABR* gene groups in WNT, SHH, group 3, and group 4 are: (1) *GABRA1, GABRB2*, and *GABRG2*, which code for α1, β2, and γ2 subunits, respectively; and (2) *GABRA2, GABRB1*, and *GABRG1*, which code for α2, β1, and γ1 subunits, respectively. In group 3 there is a set of *GABR* genes that exhibit high expression and have a significant correlation in expression: *GABRA5, GABRB3*, and *GABRG2 or GABRAG3*, which code for α5, β3, γ2 and γ3 subunits, respectively.

**Fig. 2:**
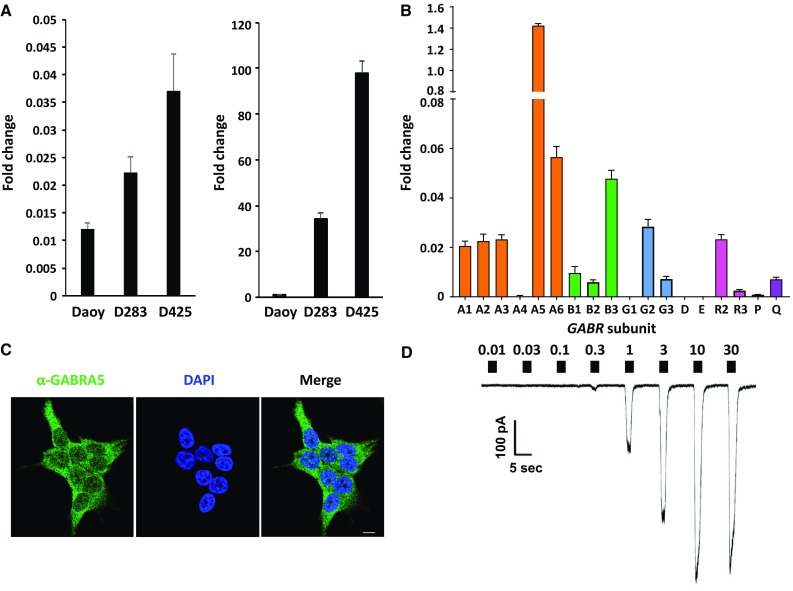
*MYC* and *GABR* expression in medulloblastoma and evidence for a functional α5-GABA_A_ receptor. **a** qRT-PCR of *N-MYC* (left) and *MYC* (right) in patient-derived lines Daoy, D283, and D425. **b** qRT-PCR of *GABR* expression in D283. Data are represented as a fold-change value with respect to expression of the housekeeping gene *TBP*, TATA Box binding protein. Values in all panels are presented as the mean and standard deviation of three experiments. Primer sequences listed in Online Resource Table 1. **c** The *GABRA5* protein product (or α5 subunit) localizes to the cell membrane in patient-derived cell line D283 with diffuse staining over the plasma membrane, as visualized by immunofluorescence microscopy using an antibody specific to the α5 subunit (green). Nucleus of cells is stained with 4′,6-diamidino-2-phenylindole (DAPI). Scale bar, 10 microns. **d** Representative current trace from a whole-cell patch clamp electrophysiology recording of a D283 cell, clamped at − 60 mV. Filled boxes above the current trace denote the period of GABA exposure (2 s) and are labeled with the concentration applied (0.01–30 µM)

To investigate how benzodiazepines may impair group 3 cell viability requires use of a cell line(s) that reflects the molecular profile of group 3 patient tumors. A significant difference in expression between group 3 subtypes and other subgroups is the degree of *GABRA5* expression. Further, group 3 tumors typically have low *N-MYC* and high *MYC* expression [11]. We analyzed expression by qRT-PCR patient-derived lines Daoy, D283 and D425 for *N*/*C-MYC* and *GABRA5*. Daoy is reported as SHH subgroup derived [24], while D283 is a group 3 medulloblastoma line, *TP53*-wildtype [25], and D425 is a group 3 medulloblastoma line, *TP53*-mutated [26]. qRT-PCR reveals that Daoy, D283, and D425 have a low and similar degree of expression of *N-MYC* (Fig. 2a). Daoy has no significant expression of *MYC*. In contrast, D283 and D425 have high *MYC* expression, characteristic of some WNT and group 3 tumors. As noted, group 3 tumors have high correlative expression of *GABRA5, GABRB3*, and *GABRG2*, which cluster on chromosome/locus 15q12. In addition, group 3α patient tumors have high *GABRA1* expression. D283 has very high *GABRA5* expression, relative to other *GABRA* genes, and higher *GABRB3* and *GABRG2* than other *GABRB* and *GABRG* genes, respectively (Fig. 2b). There is a consistency in expression between group 3 tumors and D283 cells. Most likely, D283 cell line is representative of group 3β or 3γ, given the lower *GABRA1* expression detected by qRT-PCR, which is more reflective of group 3α.

### A functional α5-GABRAR in D283 cells

Immuno-staining for the α5-subunit shows diffuse staining that appears localized to the plasma membrane (Fig. 2c). To establish that D283 cells express functional GABA_A_Rs, we obtained whole-cell patch clamp recordings. If functional GABA_A_Rs were expressed on the cell surface, then its agonist GABA should elicit a concentration-dependent chloride-anion flux. For D283 cells the average maximal current, EC_50_, and Hill slope of GABA responses in D283 cells was − 480 ± 120 pA, 1.26 ± 0.05 µM, and 1.37 ± 0.07 respectively (where *n* = 8) (Fig. 2d), demonstrating a concentration-dependent chloride-anion flux commensurate with GABA concentration. The electrophysiology recordings also provide insight into GABA_A_R subtype, chloride-anion flux rate, and number of functional receptors per D283 cell. The low GABA EC_50_ of the native GABA-sensitive receptor in D283 cells is consistent with expression of a α_5_β_3_γ_2_ or γ_3_-like GABA_A_R, supported by qRT-PCR analysis as well as *GABR* expression in group 3 tumors. The basal chloride-anion efflux rate is ~ 2 × 10^9^ ions/s, consistent with the rate of recombinant expressed GABA_A_R. We estimate that there are ~ 1000 functional α5-GABA_A_Rs per D283 cell.

### Benzodiazepines are potent α5-GABAAR modulators

**Fig. 3:**
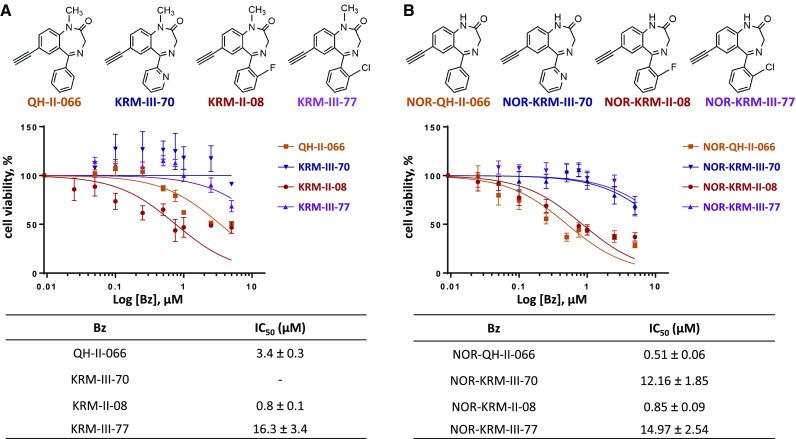
Cell viability impaired by α5-preferring benzodiazepines. Chemical structures of α5-selective benzodiazepines (**a**) and NOR variants (**b**) tested (top), dose–response curves from MTS cell proliferation assay at 48 h (middle) presented as semi-log plots and derived IC_50_ values (bottom)

We screened benzodiazepines to identify aspects of the chemical structure critical to potency (Fig. 3). All benzodiazepines examined were synthesized to be α5-GABA_A_R preferring and differed chemically at R^1^, endocyclic 2′, or exocyclic R^2′^. The most potent benzodiazepines have a hydrogen at R^1^ and no modification at the endocyclic 2′ or exocyclic R^2′^ (NOR-QH-II-066) or fluoride at the exocyclic R^2′^ (KRM-II-08 and NOR-KRM-II-08). Benzodiazepines with a larger halide (e.g. chloride) at exocyclic R^2′^ (KRM-III-77 and NOR-KRM-III-77) are poorer ligands for α5-GABA_A_R. The 2′-F at the exocyclic R^2′^ on KRM-II-08 may form a better three-centered hydrogen bond in the α5-GABA_A_R binding site, consistent with in silico modeling [27, 28]. We note an apparent increase in cell growth for KRM-III-70, which has an exocyclic nitrogen at R^2′^. This benzodiazepine may bind to an alternative target such as the peripheral benzodiazepine channel TSPO (see below), which could enhance mitochondrial function and cell proliferation.

### Benzodiazepine enhances chloride-anion efflux

**Fig. 4:**
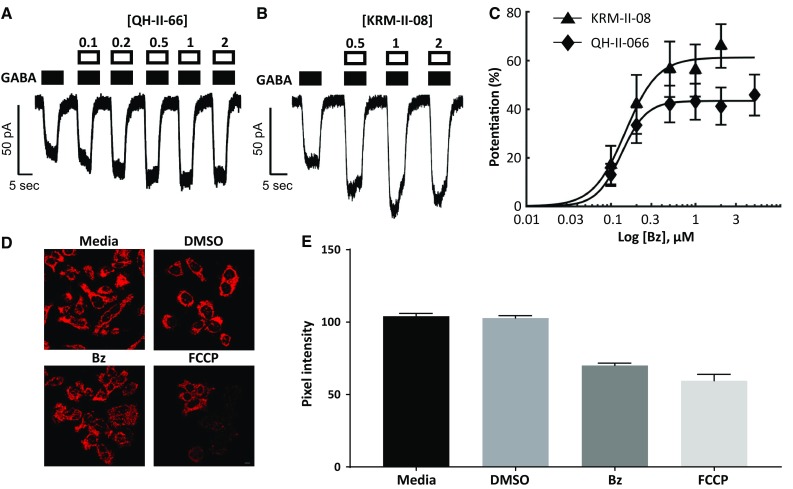
Early chemical and physiological response of group 3 medulloblastoma cells treated with α5-selective benzodiazepines. **a**, **b** D283 cells, clamped at − 60 mV, responses to GABA by α5-selective benzodiazepines QH-II-066 (**a**) and KRM-II-08 (**b**). Filled boxes above current trace denote duration of GABA application. Open boxes denote the period of benzodiazepine exposure and are labeled with the concentration applied. **c** Both QH-II-066 and KRM-II-08 (Bz) show enhanced submaximal (EC_5_–EC_10_) responses in a concentration-dependent manner: PC_50_: 43 ± 7 versus 61 ± 9, Hill slope 2.7 ± 5 versus 2.9 ± 5 and PC_50_ 0.13 ± 0.09 versus 0.14 ± 0.07 µM, respectively. The effects of QH-II-066 and KRM-II-08 were not significantly different from one another (*p* > 0.05, Student’s *t*-test). **d** Fluorescence microscopy imaging of live D283 cells stained with tetramethylrhodamine ethyl ester (TMRE) following a 10-min treatment with dimethyl sulfoxide (DMSO; 0.125%), carbonyl cyanide 4-(trifluoromethoxy) phenylhydrazone (FCCP, 20 µM), or KRM-II-08 (Bz) (0.7 µM). Media alone had no DMSO. Peak: λ_ex_, 549 nm; λ_em_, 575 nm. **e** Quantitation of TMRE staining with the Leica Application Suite X (LAS X) software platform. Data are presented as standard error from mean of thirty or more cells (media, n = 30; DMSO, n = 43; KRM, n = 39; FCCP, n = 35). Scale bar in panel (**d**) image for FCCP is 10 microns

We pursued for greater analysis QH-II-066 and KRM-II-08, which have IC_50_ values of 3.4 ± 0.3 and 0.8 ± 0.1 µM, respectively. Whole-cell recordings were obtained of the effect of these benzodiazepines on GABA_A_R function in D283 cells (Fig. 4a–c; Online Resource 3). QH-II-066 and KRM-II-08 enhanced EC_10_ responses in a concentration-dependent manner: PC_50_: 43 ± 7 versus 61 ± 9, hill slope 2.7 ± 5 versus 2.9 ± 5 and PC_50_ 0.13 ± 0.09 versus 0.14 ± 0.07 µM, respectively. The high apparent affinity for GABA in D283 cells is consistent with the presence of functional α5-GABA_A_Rs. The EC_50_ values for QH-II-066 and KRM-II-08 are similar in all assays performed, *p* > 0.05 Student’s *t*-test, in contrast to their IC_50_ values. In all cases, the modulation peaks below 2 µM and has a maximum effect of ~ 50%.

Given the lower IC_50_ of KRM-II-08 as well as greater solubility than QH-II-06 and its potential for future therapeutic use, we assessed its hepatocyte toxicity profile. LD_50_ for KRM-II-08 in vitro is > 100 µM, tested in two cell lines (HEK293 and HEPG2) (Online Resource 4). KRM-II-08 is non-toxic until the concentration is less than or equal to 100 µM, a concentration higher than IC_50_ and EC_50_ values.

### Benzodiazepine induces changes in mitochondria

Since we expected that benzodiazepine binding to GABA_A_R in group 3 cells might alter ionic flux rapidly assuming exogenous GABA is ≥ 0.3 µM, we examined changes to mitochondria and its membrane potential. Staining for GABA_A_R at the plasma membrane remains similar and constant in DMSO and KRM-II-08 treated cells as well as untreated cells over 48 h (Online Resource 5), suggesting that the receptor remains intact and possibly then functional. We examined changes in mitochondrial morphology using the cationic stain tetramethylrhodamine ethyl ether (TMRE), which is taken-up by functioning mitochondria. Ten-minutes following benzodiazepine treatment, mitochondria have undergone fission but continue to take-up TMRE (Fig. 4d). Fission of mitochondria is not observed in DMSO but is when a protonophore, carbonyl cyanide-4-(trifluoromethoxy)phenylhydrazone (FCCP) is added.

FCCP disrupts mitochondrial ATP synthesis, depolarizing mitochondria or causing loss of ΔΨm [29]. FCCP is used as a positive control for monitoring change in mitochondria membrane potential, as it causes reduced TMRE staining. We quantified the degree of TMRE staining of thirty or more cells in all treatment groups (Fig. 4e). KRM-II-08 causes a depolarization of mitochondrial membrane potential within 10 min, but not DMSO.

There is, as noted above, a chloride-anion efflux in D283 cells commensurate with benzodiazepine administration that mediates membrane depolarization. Present in the outer mitochondrial membrane is the peripheral benzodiazepine metabotropic receptor TSPO to which diazepam has reported to bind and whose activity can reduce mitochondrial membrane potential [30]. We tested if TSPO agonist emapunil has an effect on viability of Daoy and D283 cells to determine if the observed potency of KRM-II-08 was a consequence of its binding to TSPO (Online Resource 6). Emapunil does not impair viability of Daoy or D283 cells. This observation supports the contention that the primary and effective binding site of KRM-II-08 that induces apoptosis is not TSPO.

### p53 response to benzodiazepine

Previously we demonstrated that benzodiazepines were capable of impairing group 3 cell viability, including of cell line D425, which has a *TP53* exon 4 single-nucleotide polymorphism (R72P) that has been reported to impact the apoptotic response to some types of stress [31]. Since D425 response to the benzodiazepines tested was not impacted by the *TP53* polymorphism, this supports p53 not being critical to the cell death response. However, this point mutation may not impair all functions of p53 and the apoptotic response of some types of stress are not impacted [32]. We also previously observed that benzodiazepines were capable of sensitizing group 3 cells to either radiation or a chemotherapeutic, abrogated by a p53 knockdown [17], which supports the role of p53 in the apoptotic response mediated by benzodiazepines.

**Fig. 5:**
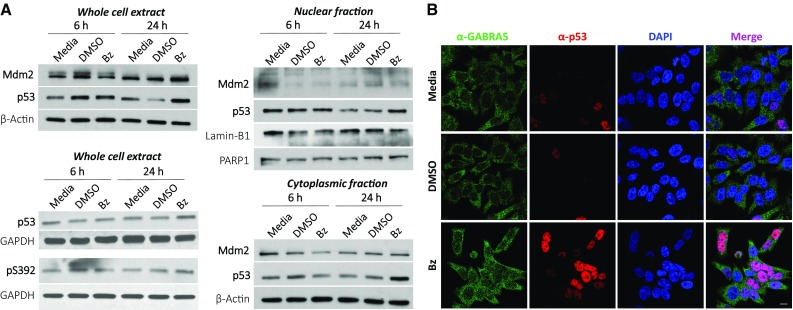
Contribution of p53 to response of α5-preferring benzodiazepine KRM-II-08. **a** Western blot of Mdm2 and p53 at 6 and 24 h post-treatment with KRM-II-08. Western blots of whole cell (top), cytoplasmic (middle), and nuclear (bottom) extracts. Loading controls for blots are beta-actin, Lamin-B1, and/or PARP1. Western blot of p53 using antibodies that recognize the protein regardless of post-translational modification and specific to phosphorylation of p53 Serine392 (pS392). GAPDH is the loading control. **b** Immunofluorescence microscopy imaging of D283 cells at 24 h following incubation with media alone, DMSO, or KRM-II-08 (Bz, 0.8 µM). Cells were stained using antibodies specific to α5 (green) and p53 (red). Nucleus of cells were stained with 4′,6-diamidino-2-phenylindole (DAPI). Scale bar in bottom, right image is 10 microns

Since p53 appears to play a critical role in the stress-response to benzodiazepine mediated chloride-anion efflux and its DNA-binding domain contributes to this role, we examined the impact of the benzodiazepine KRM-II-08 on expression of genes that participate in the *PTEN-TP53-AKT-MDM2* signaling axis [33]: PI3K molecules (Class I regulatory and catalytic subunits, Class II, and Class III); serine/threonine kinases *AKT1*, *AKT2*, and *AKT3*; *PTEN*, the phosphatase which negatively regulates the PI3K/Akt signaling pathway, stabilizes p53, and whose expression is regulated by p53; and *MDM2*, which codes for the E3 ubiquitin ligase that functions as a negative regulator of p53. We examined changes in expression of these genes as well as *TP53* in D283 cells at 6 and 24 h post-incubation with KRM-II-08. *MDM2*, *PTEN*, *AKT1*-*3* as well as *TP53* are upregulated in KRM-II-08 treated cells, which is benzodiazepine-specific, as DMSO causes no change in *TP53* and *PTEN* levels while *MDM2 and AKT1-3* expression are down-regulated (Online Resource 7). Of *PI3K* genes, only Class I catalytic and regulatory subunits PI3CA and PIK3R1, respectively, are significantly upregulated. *MDM2* protein levels also appear to increase moderately between 6 and 24 h post-KRM-II-08 treatment, while p53 levels increase significantly at 24 h and in both nuclear and cytoplasmic fractions (Fig. 5a). As well as an increase in p53 by Western blot, we observe an increase in p53 by immunofluorescence with the most intense staining in the nucleus (Fig. 5b).

Previously we observed that a less potent benzodiazepine studied here (see QH-II-066, IC_50_ 3.4 µM), caused cell cycle arrest [17]. We therefore repeated an analysis on the cell cycle of D283 cells of the more potent benzodiazepine KRM-II-8 (IC_50_ 0.8 µM). KRM-II-08 does not arrest the cell cycle of D283 cells at 24 or 48 h (Online Resource 8). This suggests that the less potent benzodiazepine tested earlier may have a secondary or ‘off-target’ effect in group 3 cells. However, arresting the cell cycle is not critical to benzodiazepine-mediated apoptosis.

### Activation of cell death

There are early changes in mitochondrial structure–function, which may precipitate events that result in D283 cell death. However, cell death is not immediate and may require p53 transcriptional activity as well as its migration to the cytoplasm. We initially examined whether D283 cells underwent senescence. Analysis of senescence-associated β-galactosidase of DMSO and KRM-II-08 treated cells reveals that in 48 h at most ~ 12% of cells may be undergoing senescence (Online Resource 9), which does not account for loss in cell viability observed using KRM-II-08. We subsequently utilized an immune-blotting approach to identify change in levels and/or post-translational modification of proteins that have a role in apoptosis in D283 cells incubated with DMSO or KRM-II-08. There is a modest change in the degree of p53 post-translational modification, specifically phosphorylation of Serine392 (pS392) (Online Resource 10). We confirmed by Western that p53 is phosphorylated at S392 (Fig. 5a). While S392 exhibits increased phosphorylation in KRM-II-08 treated cells, it’s also modified in control cells (DMSO and media). Thus, pS392 appears to be a constitutive modification in D283 cells.

Since senescence did not account for the death of most benzodiazepine treated cells, we examined by immunofluorescence KRM-II-08 treated D283 cells for change in amount and/or localization of pro-apoptotic Bcl-2 family members Bax, Puma, Bcl-2, Bcl-xL, and BAD [34]. Only BAD protein exhibits a change in intensity detected by immunofluorescence in KRM-II-08 treated D283 cells and there is a slight increase in BAD protein levels between 6 and 24 h (Online Resource 11). It’s been reported that BAD and p53 do complex at mitochondria to induce apoptosis [35].

## Discussion

In medulloblastomas we find that *GABR* genes are expressed in all subgroups. Interestingly, we find that WNT subgroup patients appear to have a unique shared *GABR* expression signature. In contrast, not all SHH subgroup patients have a shared *GABR* expression signature, however, there is a specific subset of SHH subgroup patients (the SHHγ subtype) that do. These observations may be connected to activation of distinct signaling pathways in these subgroups and warrant further analysis. In this study, we have also explored in detail the *GABR* signature in group 3 patients and the functional and therapeutic implications of the signature. We report that the group 3 cell line D283 has a functional α_5_β_3_γ_2_ or γ_3_-like GABA_A_R and have shown the physical, chemical, and molecular changes to group 3 cells that precede their death, as a consequence of α5-GABA_A_R preferring benzodiazepine enhancing the activity of GABA.

In a non-neural cell, GABA_A_R may polarize a cell by creating a chloride-anion flux, which may drive cell proliferation [36]. Alternatively, a chloride-anion flux may elicit a stress-response, if it significantly perturbs ionic homeostasis [37]. We have shown that the α_5_β_3_γ_2_ or γ_3_-like GABA_A_R in group 3 cells mediates a significant chloride-anion efflux to depolarize mitochondria, when an α5-GABA_A_R preferring benzodiazepine binds in the presence of GABA, such that the cell activates a stress-response involving p53 and that this sustained effect induces apoptosis.

In our analysis of p53 response to benzodiazepine, we find that p53 is constitutively phosphorylated at S392. S392 phosphorylation stabilize p53’s tetrameric state, which decreases its turnover and increases its DNA-binding affinity [38]. We have not examined the oligomeric state of cytoplasmic p53, but it may serve a role in determining its cytoplasmic function that includes an increased affinity for the pro/anti-apoptotic protein BAD. In addition, S392 hyperphosphorylation is correlated with poor prognosis in several cancers [39–41], and this may be the case in medulloblastoma.

### Conclusion

Altered GABA levels or high expression of GABA_A_R subunits has been observed in pediatric as well as adult cancers [42–46]. Ion channels have potential to be promising anti-cancer therapeutic targets [47] and a significant number of FDA approved drugs target GABA_A_Rs. α5-GABA_A_R preferring benzodiazepine KRM-II-08 is like other benzodiazepines predicted to be non-toxic and capable of crossing the blood–brain barrier. While we have shown in cell culture that KRM-II-08 is non-toxic, further testing in vivo is warranted. KRM-II-08 may be an effective therapeutic to be included in treating medulloblastoma and other cancers. Moving in this direction will require more extensive studies in an appropriate animal model, possibly exploring impact of administration of benzodiazepine in combination with radiation and/or other therapeutics.

## Electronic supplementary material

Below is the link to the electronic supplementary material.

Supplementary material 1 (DOCX 3532 KB)
