# Supplementary material for: Modulating native GABAA receptors in medulloblastoma with positive allosteric benzodiazepine-derivatives induces cell death
Source: J Neurooncol. 2019 Feb 6;142(3):411–22. doi: 10.1007/s11060-019-03115-0 (PMC6478651; doi:10.1007/s11060-019-03115-0)
Supplement: Supplementary file 1 — Supplementary material 1 (DOCX 3532 KB) [file 11060_2019_3115_MOESM1_ESM.docx]

**SUPPLEMENTARY MATERIAL**

**Figures**

**
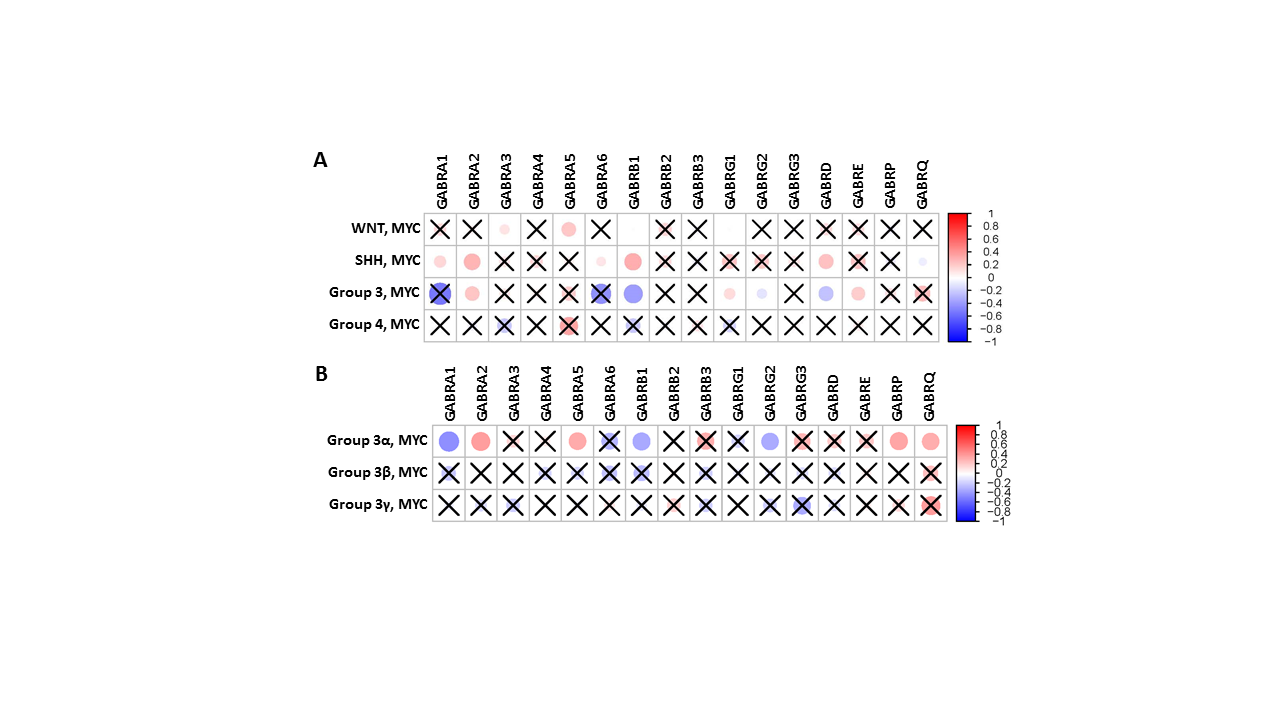
**

**Online Resource 1. GABA_A_ receptor subunit (*GABR*) and *MYC* correlation in gene expression in 763 medulloblastoma tumors.** (A) Correlogram of *MYC* with *GABR* gene expression across all four medulloblastoma subgroups. (B) Correlogram of *MYC* with *GABR* gene expression within group 3 subtypes (α, β, and γ). Positive correlation values are indicated in red and negative values in blue. Correlation values not marked by an "X" are statistically significant, using a correlation test (Spearman’s) at *p*-value < 0.01. See Online Resource Tables II and III for values. Normalized gene expression data for sixteen *GABR* genes and *C-MYC or MYC* from 763 primary resected medulloblastoma specimens was used [1]. Correlation analyses among the *GABR* genes and *MYC* were performed using Spearman’s correlation. Correlograms were used to summarize the correlations using the ‘corrplot’ R package.

**
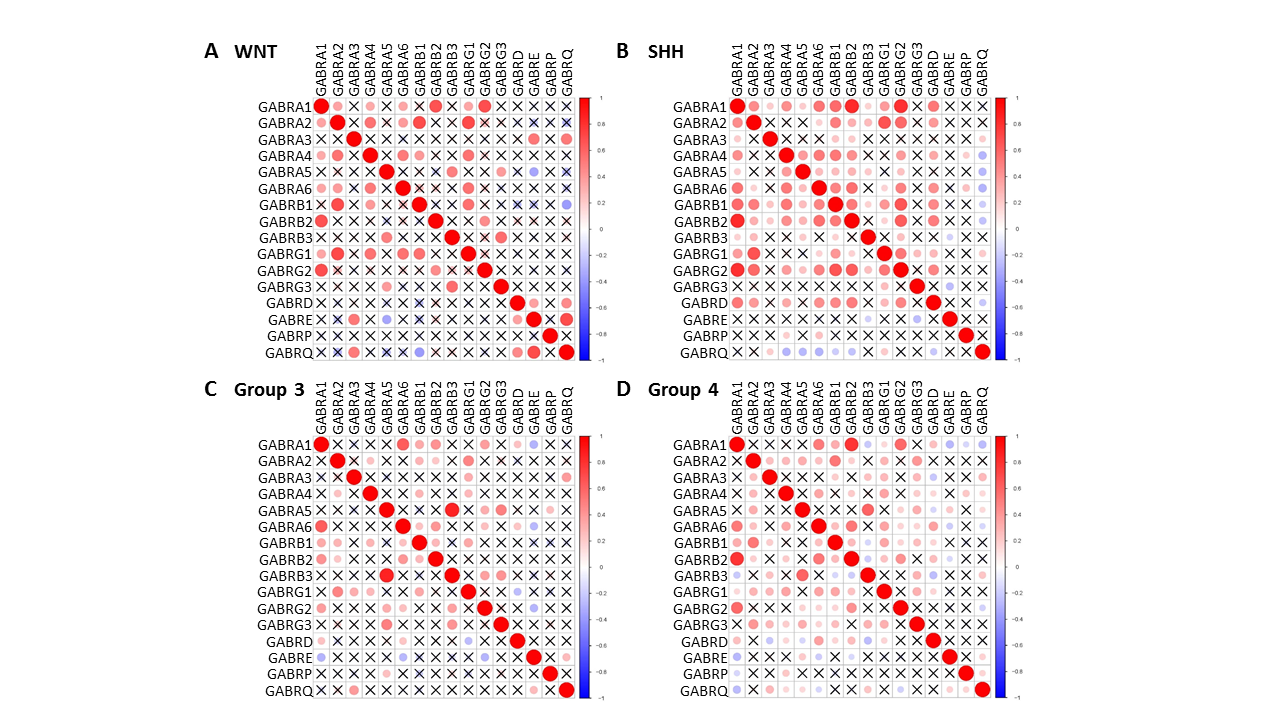
**

**Online Resource 2. GABA_A_ receptor subunit (*GABR*) correlation in gene expression in 763 medulloblastoma tumors.** Correlograms of *GABR* gene expression by subgroup: (A) WNT, (B) SHH, (C) group 3, and (D) group 4. Positive correlation values are indicated in red and negative values in blue. Correlation values not marked by an "X" are statistically significant, using a correlation test at *p*-value < 0.01.

**
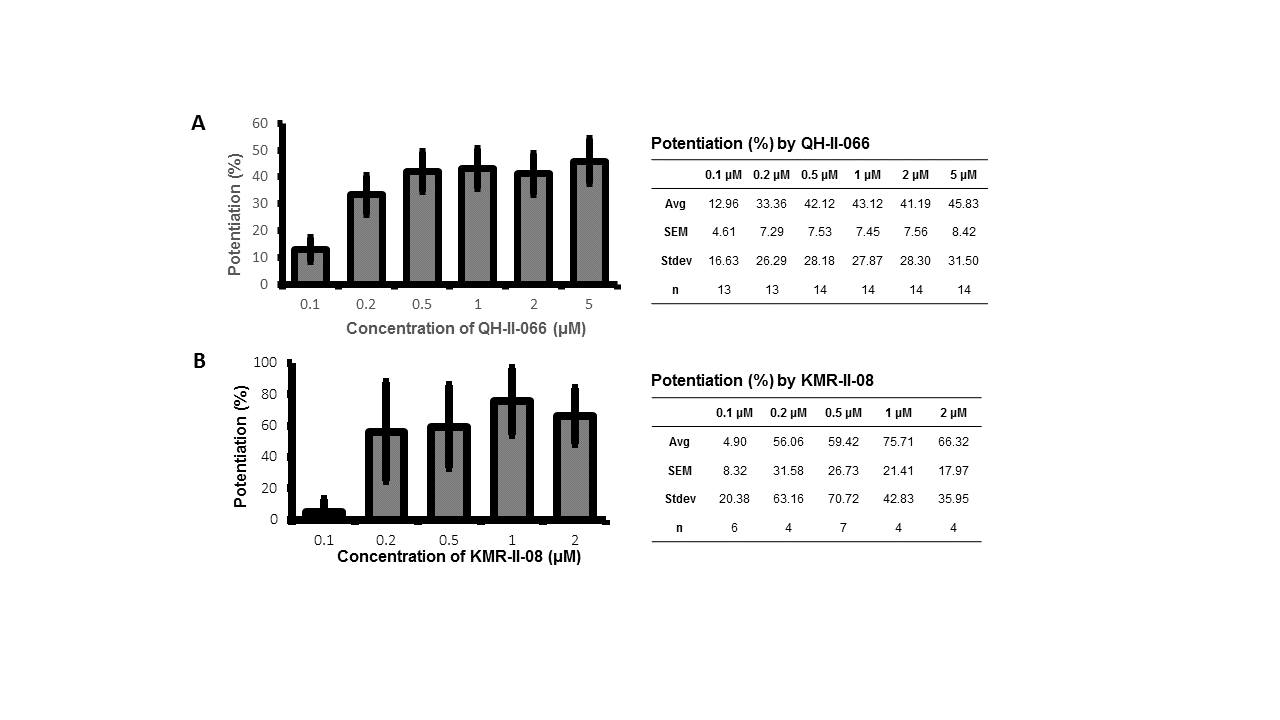
 Online Resource 3. Data of whole-cell patch clamp recordings from D283 cells.** Patient-derived medulloblastoma cell line D283, clamped at -60 mV, responses to GABA by α5-selective benzodiazepines QH-II-066 (A) and KRM-II-08 (B). Both benzodiazepines show enhanced submaximal (EC_5_-EC_10_) responses in a concentration-dependent manner. The effects of QH-II-066 and KRM-II-08 were not significantly different from one another (*p*>0.05, Student’s *t*-test). Whole-cell patch-clamp recordings were performed on D283 cells using methods similar to those previously described [2]. GABA concentration-response assays were performed by exposing each whole-cell patch to increasing concentrations of GABA for 2 sec, with an 8 sec washout between concentrations. Recordings were baseline corrected and analyzed in MATLAB (MathWorks). Peak currents (*I*) were measured from GABA exposures and fitted using least-squares nonlinear regression analysis based on the Hill equation: *I = I_max_ *[A]^nH^/(EC_50_^nH^ + [A]^nH^)*, where *I* is current peak amplitude, *I_max_* is maximum current amplitude, *EC_50_* is the GABA concentration producing the half-maximal response, *A* is agonist concentration, and *nH* is the Hill coefficient. GABA concentration-response assays were individually fitted to the Hill equation for each whole-cell recording. The maximum peak current, EC_50_, and Hill coefficient were estimated based on averaged values for the receptor and are reported as mean ± standard error of the mean (SEM). Benzodiazepine concentration response relationships were constructed for QH-II-066 (0.1 – 2 µM) and KRM-II-08 (0.1 – 5 µM). For each D283 cell, reference EC_10_ GABA response was established (C) before determining peak response to co-application of benzodiazepine and GABA EC_10_ (M). Percent potentiation (P) was calculated using equation: *P=100x(M-C)/C*. Potentiation was fitted using least-squares nonlinear regression analysis based on equation: *P = P_max_ *[Bz]^nH^/(PC_50_^nH^ + [Bz]^nH^)*, where *P* is potentiation, *P_max_* is maximum potentiation, *PC_50_* is benzodiazepine concentration producing the half-maximal effect, *Bz* is the benzodiazepine concentration, and *nH* is the Hill coefficient. Concentration-Potentiation relationships were individually fitted to the Hill equation for each whole-cell recording. The maximum potentiation, *PC_50_*, and Hill coefficient were estimated based on averaged values for the receptor and are reported as mean ± standard error of the mean (SEM). Whole-cell currents were recorded at ‑60 mV, filtered at 100 Hz, and sampled at 200 Hz with a MultiClamp 700B amplifier and DigiData 1322A digitizer (Molecular Devices).

**
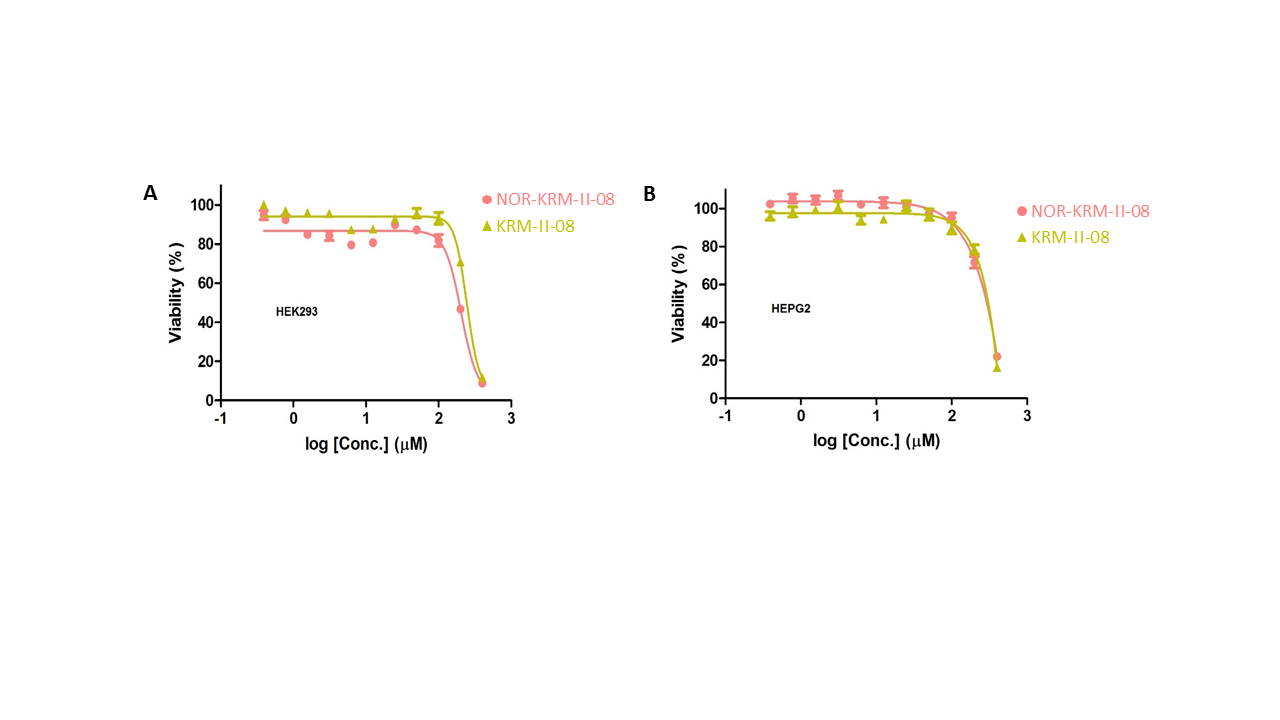
 Online Resource 4. Cytotoxicity assay of benzodiazepines**. (A) Measurement of cytotoxicity of KRM-II-08 and the NOR variant using human embryonic kidney 293TH or HEK293 (A) and human liver hepatocellular carcinoma or HEPG2 (B) cell lines. In both the cell lines the LD_50_ values for KRM-II-08 and the NOR variant are greater than 100 μM. Hence, KRM-II-08 is safe and non-toxic until the concentration is less than or equal to 100 μM. Cytotoxicity of benzodiazepines was tested as described previously [3, 4]. HEK293T and HEPG2 cell lines were purchased (ATCC) and cultured in 75 cm^2^ flasks (CellStar). Cells were grown in DMEM/High Glucose media (Hyclone) to which non-essential amino acids (Hyclone), 10 mM HEPES (Hyclone), 5 x 106 units of penicillin and streptomycin (Hyclone), and 10% of heat inactivated fetal bovine serum (Gibco) were added. Cells were harvested using 0.05% Trypsin (Hyclone), washed with PBS, and dispensed into sterile white, optical bottom 384-well plates (NUNC). After two hours, small molecule solutions were transferred with a Tecan Freedom EVO liquid handling system equipped with a 100 nL pin tool (V&P Sci.). The controls were 3-dibutylamino-1-(4-hexyl-phenyl)-propan-1-one (25 mM in DMSO, positive control) and DMSO (negative control). The cells were incubated for 48 hours followed by the addition of CellTiter-Glo™, a luminescence-based cell viability assay (Promega). All luminescence readings were performed on a Tecan Infinite M1000 plate reader. The assay was carried out in quadruplet with three independent runs. The data were normalized to the controls and analyzed by nonlinear regression (GraphPad Prism).

**
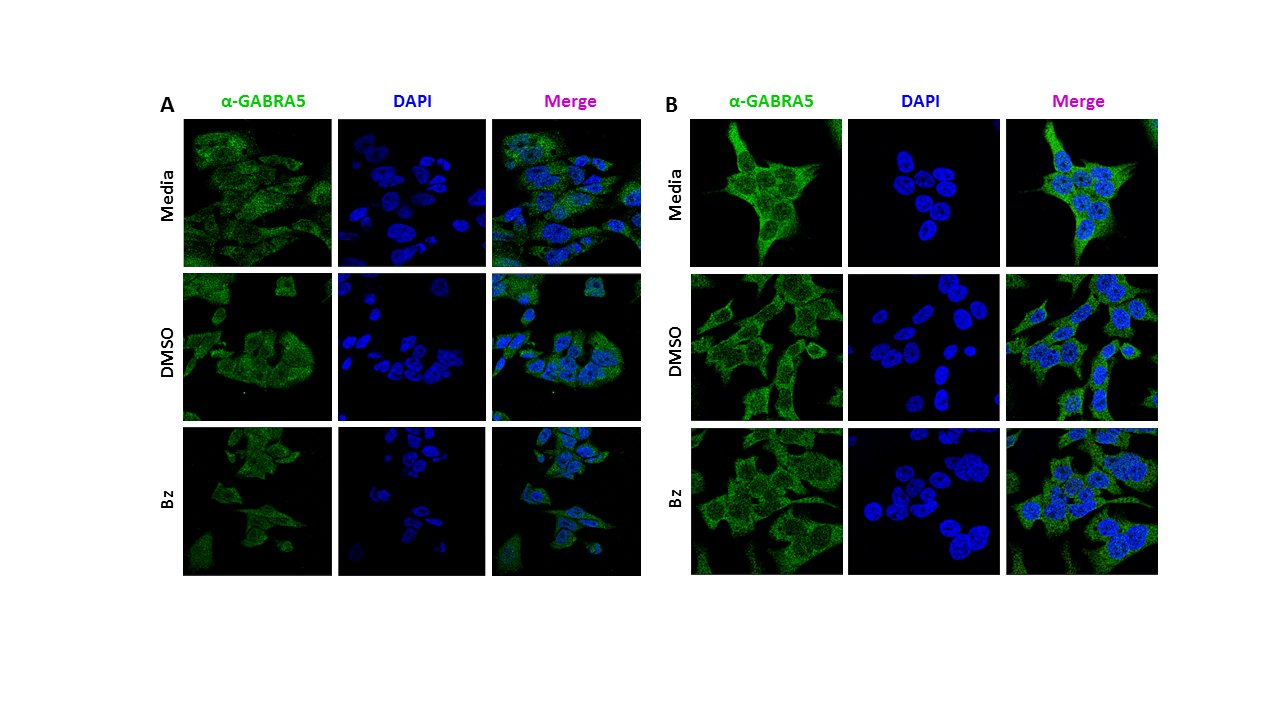
**

**Online Resource 5. Assessment of change in native GABA_A_R in D283 cells over time following exposure to KRM-II-08.** D283 cells were untreated (media), treated with DMSO, or treated with KRM-II-08 (Bz) for 6 (A) and 24 (B) hours and then stained for imaging. There is no detectable change in degree or localization of staining for protein product of *GABRA5* (green).

**
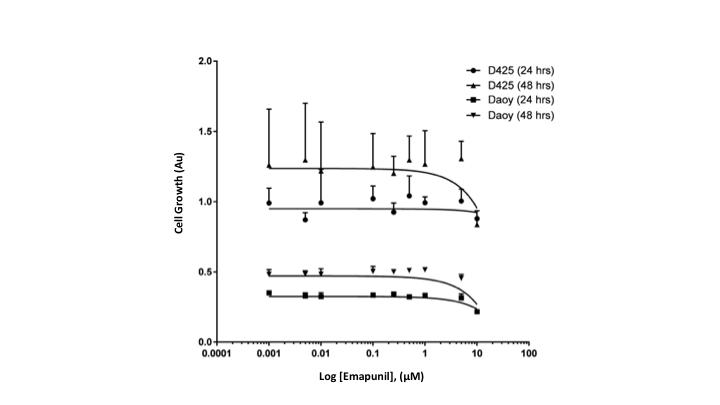
**

**Online Resource 6. Examination of the effect of emapunil on medulloblastoma cells.** Emapunil at different concentrations was incubated with medulloblastoma cells Daoy and D425 for 24 and 48 hours and the cell viability tested as described in Materials & Methods section “cell proliferation assay”.

**
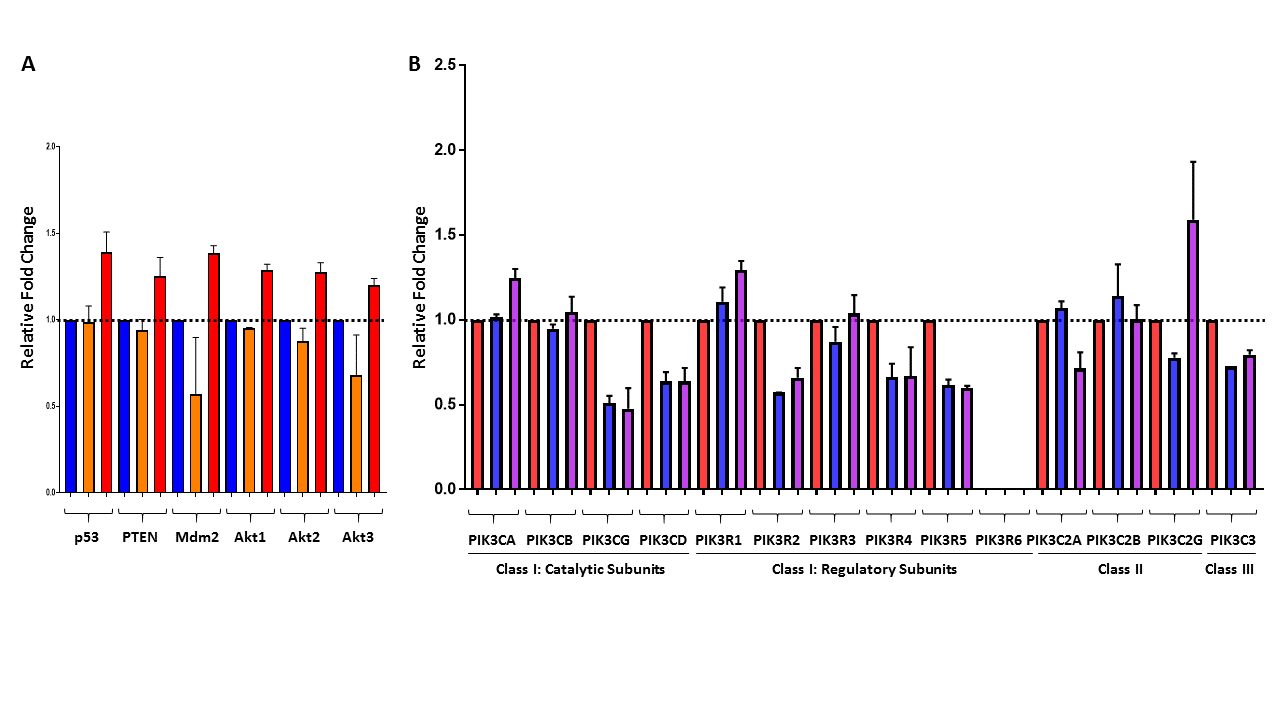
 Online Resource 7. Quantitative RT-PCR of gene expression in D283 cells following KRM-II-08 exposure.** (A) *TP53*, *PTEN*, *MDM2* and *AKT1-3* expression in D283 cells exposed to media (blue column), DMSO (orange column), or KRM-II-08 (red column). (B) *PI3K* subunit expression in D283 cells exposed to media (red column), DMSO (blue column, or KRM-II-08 (purple column).

**
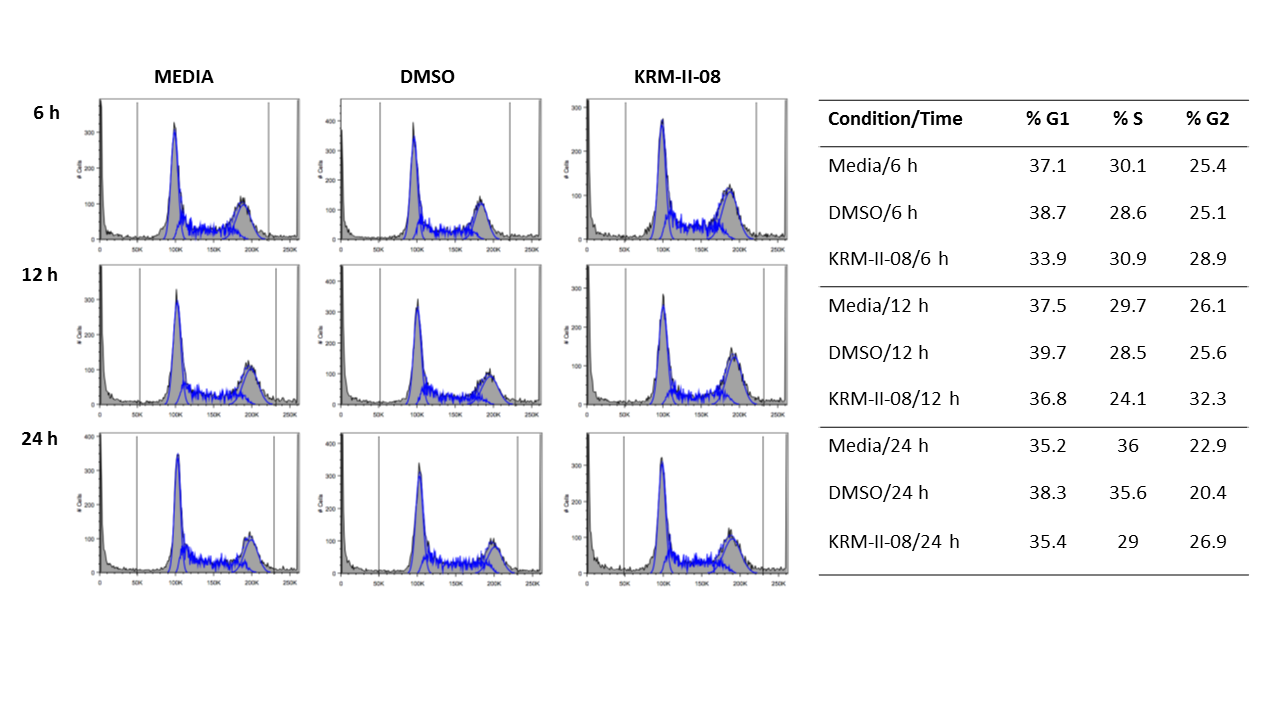
 Online Resource 8. KRM-II-08 does not alter cell cycle.** Shown is fluorescence-activated cell sorting or FACS data of D283 cells untreated or media control (left three panels), treated with DMSO (middle three panels), or α5-preferring benzodiazepine KRM-II-08 (right three panels) at 6, 12, and 24 hours. Table is provided of peak values at G1, S, and G2 phases of the cell cycle for the three experimental groups. D283 cells were plated at 140,000 cells/mL in DMEM/F12 without phenol red or antibiotics and with 20% fetal bovine serum (FBS). Following an overnight incubation at 37°C; 5% CO2, KRM-II-08 or the equivalent volume of DMSO was added from 4x stock solutions to a final concentration of 0.7 μM KRM-II-08. Media controls and treated cells were incubated at 37°C; 5% CO2. After 6, 12 or 24 hours, cells were harvested, washed with cold 1x PBS, resuspended in 70% ethanol and stored at -20°C overnight. After removal of ethanol, DNA was stained with propidium iodide (PI)/RNase staining buffer (BD Biosciences) according to the manufacturer’s instructions. Cell cycle analysis was performed using a BD FASCCANTO II flow cytometer and FlowJo analysis software.

**
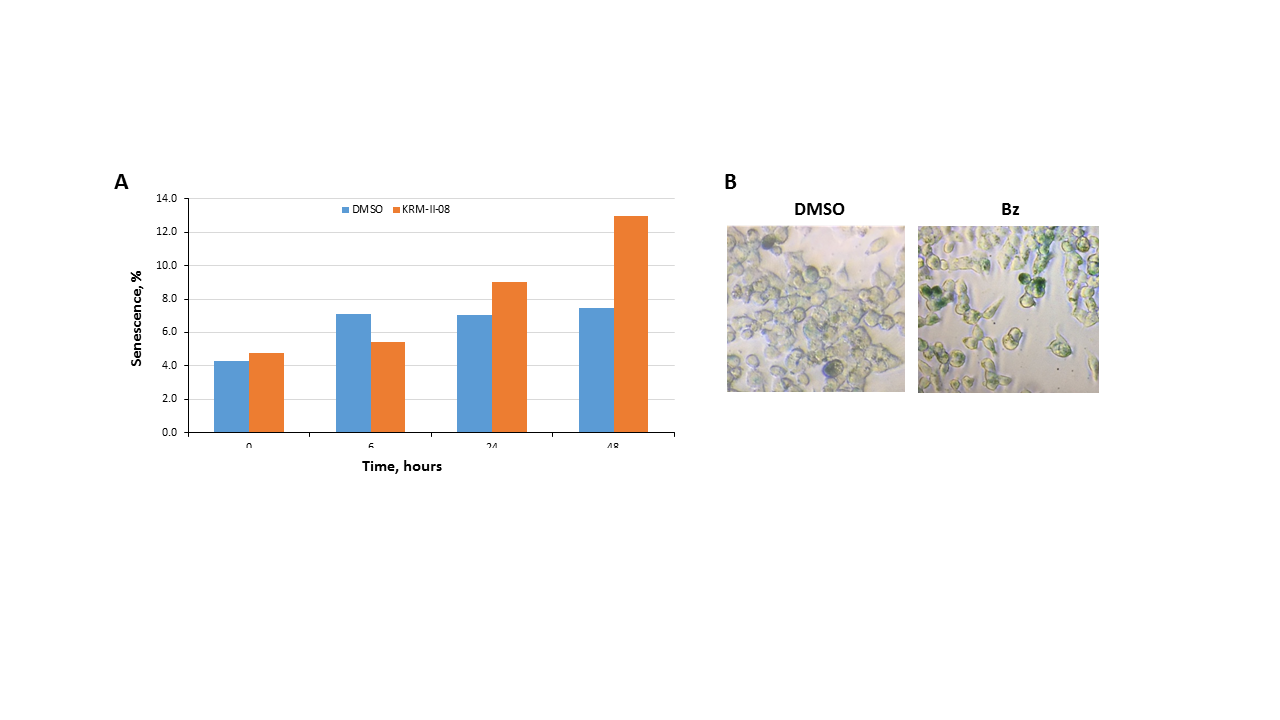
 Online Resource 9. Examination of senescence D283 cells treated with KMR-II-08**. (A) Time course of D283 cells treated with either DMSO (blue) or 0.1 μM KRM-II-08. (B) Cells treated with DMSO or KRM-II-08 were stained for β-galactosidase, a marker for senescence, which was quantified.

**
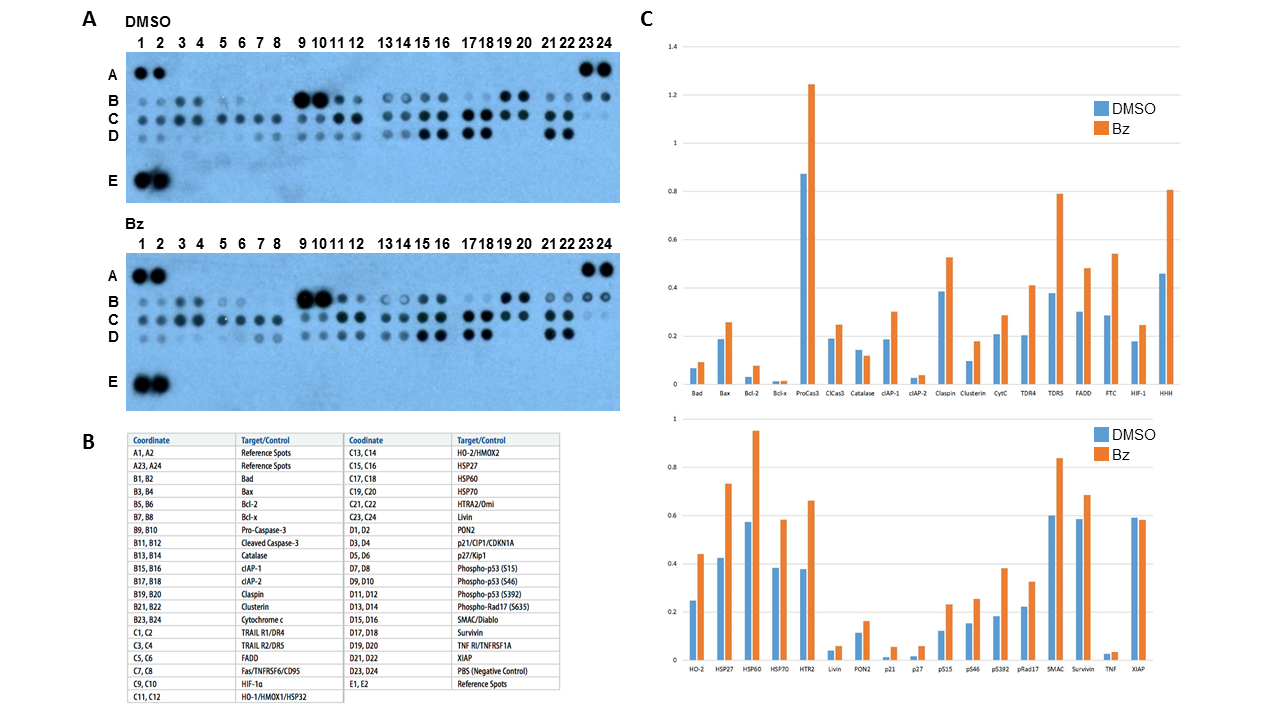
Online Resource 10. Apoptosis proteome profiler array assay.** (A) Raw data of D283 cells treated with DMSO (top image) or 0.7 µM KRM-II-08 (bottom image) for 24 hours. (B) Guide to wells labeled in two images in panel “A”. (C) Quantification of gels in panel “A”. Relative levels of apoptosis-related proteins from D283 cells treated for 24 hours with either DMSO or 0.7 µM KRM-II-08 were evaluated using the Human Apoptosis Array Kit (R&D Systems, ARY009). D283 treated and control lysates (300 µg) were incubated overnight with human apoptosis arrays composed of capture and control antibodies targeting representative apoptosis pathway proteins immobilized in duplicate on nitrocellulose membranes. The arrays were washed to remove unbound proteins. Bound proteins were incubated with a cocktail of biotinylated antibodies followed by Streptavidin-HRP and chemiluminescent detection. Signals are quantitated and identified using the array key.

**
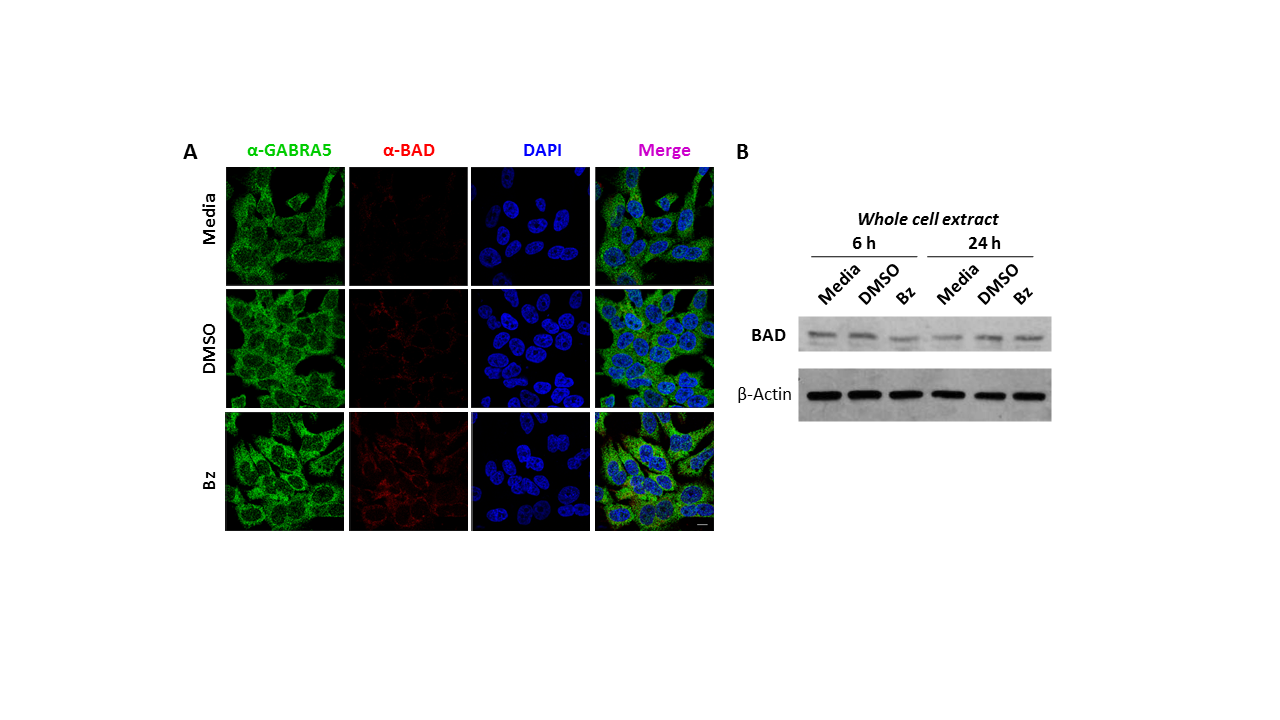
**

**Online Resource 11. Activation of apoptosis*.*** (A) Immunofluorescence microscopy imaging of D283 cells at 24 h following incubation with media alone, DMSO, or KRM-II-08 (Bz, 0.8 μM). Cells were stained using antibodies specific to α5 (green) and the protein BAD (red). Nucleus of cells were stained with 4′,6-diamidino-2-phenylindole (DAPI). Scale bar in bottom, right image is 10 microns. (B) Western blot of protein BAD at 6 and 24 h post-incubation with KRM-II-08 (Bz). Loading control for blot is β-actin.

**Online Resource Tables**

**Table 1.** RT-PCR primers used in the study.

| **Gene** | **Primer** | **Gene** | **Primer** |
| --- | --- | --- | --- |
| *GABRA*^1^ | F: GGATTGGGAGAGCGTGTAACC | *GABRR3^1^* | F: TGATGCTTTCATGGGTTTCA |
|  | R: TGAAACGGGTCCGAAACTG |  | R: CGCTCACAGCAGTGATGATT |
| *GABRA2*^1^ | F: GTTCAAGCTGAATGCCCAAT | *TBP^1^* | F: GAGCTGTGATGTGAAGTTTCC |
|  | R: ACCTAGAGCCATCAGGAGCA |  | R: TCTGGGTTTGATCATTCTGTAG |
| *GABRA3*^2^ | F: AAGGTGGGAGCCAGTTCTAC | *C-MYC^3^* | F: CAA ACC TCC TCA CAG CCC ACT |
|  | R: CAGGCTGGCAGAGCTAGT |  | R: TTC GCC TCT TGA CAT TCT CCT C |
| *GABRA4*^1^ | F: TTGGGGGTCCTGTTACAGAAG | *N-MYC^3^* | F: CATTCACCATCACTGTGCGT |
|  | R: TCTGCCTGAAGAACACATCCA |  | R: GCATCCTCACTCTCCACGTA |
| *GABRA5*^1^ | F: CTTCTCGGCGCTGATAGAGT | *Cyclin D1^3^* | F: ACAAACAGATCATCCGCAAACAC |
|  | R: CGCTTTTTCTTGATCTTGGC |  | R: TGTTGGGGCTCCTCAGGTTC |
| *GABRA6*^1^ | F: ACCCACAGTGACAATATCAAAAGC | *TP53^3^* | F: GTCCAGATGAAGCTCCCAGA |
|  | R: GGAGTCAGGATGCAAAACAATCT |  | R: CAAGAAGCCCAGACGGAAAC |
| *GABRB1*^1^ | F: GTACAAAATCGAGAGAGTCTGGG |  | F: CAACAACACCAGCTCCTCTC |
|  | R: GCGAATGTCATATCCTTTGAGCA |  | R: TGGGCATCCTTGAGTTCCAA |
| *GABRB2*^1^ | F: GCAGAGTGTCAATGACCCTAGT | *MDM2^3^* | F: GTGCCAAGCTTCTCTGTGAAA |
|  | R: TGGCAATGTCAATGTTCATCCC |  | R: TGACACCTGTTCTCACTCACA |
| *GABRB3*^1^ | F: CAAGCTGTTGAAAGGCTACGA |  | F: GATGAAAGCCTGGCTCTGTG |
|  | R: ACTTCGGAAACCATGTCGATG |  | R: CCTGATCCAACCAATCACCTG |
| *GABRG1*^1^ | F: CCTTTTCTTCTGCGGAGTCAA | *PTEN^3^* | F: ATGTTCAGTGGCGGAACTTG |
|  | R: CATCTGCCTTATCAACACAGTTTCC |  | R: GAACTTGTCTTCCCGTCGTG |
| *GABRG2*^1^ | F: CACAGAAAATGACGGTGTGG |  | F: CACGACGGGAAGACAAGTTC |
|  | R: TCACCCTCAGGAACTTTTGG |  | R: GGTTTCCTCTGGTCCTGGTA |
| *GABRG3^1^* | F: AACCAACCACCACGAAGAAGA | *AKT1^3^* | F: CTTTCGGCAAGGTGATCCTG |
|  | R: CCTCATGTCCAGGAGGGAAT |  | R: GTACTTCAGGGCTGTGAGGA |
| *GABRD^1^* | F: ACCACGGAGCTGATGAACTT |  | F: GCCTCTGCTTTGTCATGGAG |
|  | R: AGGGCATGTAGGATTGGATG |  | R: AGCATGAGGTTCTCCAGCTT |
| *GABRE^1^* | F: TGGATTCTCACTCTTGCCCTCTA | *AKT2^3^* | F: CGGCTCTTGAGTACTTGCAC |
|  | R: GGAGTTCTTCTCATTGATTTCAAGCT |  | R: CGTCACTGATGCCCTCTTTG |
| *GABRQ^1^* | F: CCAGGGTGACAATTGGCTTAA |  | F: AGGACGTGGTCCAGAAGAAG |
|  | R: CCCGCAGATGTGAGTCGAT |  | R: TGATGGACTGGGCGGTAAAT |
| *GABRP^1^* | F: GGCCTTGCTAGAATATGCAGTTG | *AKT3^3^* | F: AACGACCAAAGCCAAACACA |
|  | R: CTTTGTTGTCCCCCTATCTTTGG |  | R: TCTCCTCTTCTTGCCTCTGC |
| *GABRR2^1^* | F: CCTAGAAGAGGGCATAGACATCG |  | F: TAATGCGGGACAAAGATGGC |
|  | R: TCCAGTAGCTGCTGCATTGTTTG |  | R: CTACTGCTCGGCCATAGTCA |

^1^ Smits A, Jin Z, Elsir T, Pedder H, Nistér M, Alafuzoff I, et al (2012) GABA-A channel subunit expression in human glioma correlates with tumor histology and clinical outcome. PLoS One 7(5):e37041. <https://doi.org/10.1371/journal.pone.0037041>

^2^ This study.

^3^ RTPrimer Database: <http://medgen.ugent.be/rtprimerdb/>

**Table 2.** Data on correlation of *GABR* and *MYC* gene expression in medulloblastoma subgroups (where *p* > 0.01 is not significant).

|  | **WNT, *MYC*** | | **SHH, *MYC*** | | **Group 3, *MYC*** | | **Group 4, *MYC*** | |
| --- | --- | --- | --- | --- | --- | --- | --- | --- |
| **Gene** | **Cor** | **p-value** | **Corr** | **p-value** | **Corr** | **p-value** | **Corr** | **p-value** |
| ***A1*** | 0.15496 | 0.75692 | 0.15435 | 1.03E-11 | -0.52809 | 0.02112 | 0.01721 | 0.2002 |
| ***A2*** | 0.07235 | 0.11244 | 0.28918 | 0.007997 | 0.220215 | 1.14E-05 | -0.0881 | 0.5517 |
| ***A3*** | 0.10097 | 0.000077 | 0.11752 | 0.1878 | 0.11036 | 0.0799 | -0.21723 | 0.4055 |
| ***A4*** | 0.03723 | 0.29632 | 0.16753 | 0.28823 | 0.089101 | 0.01223 | -0.05802 | 0.759 |
| ***A5*** | 0.21768 | 6.008E-11 | 0.08585 | 0.01271 | 0.207195 | 0.20155 | 0.3521 | 0.0702 |
| ***A6*** | -0.00661 | 0.8756 | 0.09254 | 6.0653E-08 | -0.43274 | 0.168475 | 0.0087 | 0.9567 |
| ***B1*** | 0.01133 | 0.000072 | 0.31429 | 2.79E-06 | -0.37909 | 1.68E-06 | -0.2179 | 0.9258 |
| ***B2*** | 0.17326 | 0.19651 | 0.15287 | 0.505228 | -0.05597 | 0.022408 | -0.0717 | 0.1514 |
| ***B3*** | -0.05622 | 0.02453 | -0.08407 | 0.42500 | 0.06699 | 0.21108 | 0.12454 | 0.6438 |
| ***G1*** | 0.01465 | 0.00144 | 0.23701 | 0.10874 | 0.13422 | 0.000356 | -0.1758 | 0.9042 |
| ***G2*** | -0.02795 | 0.7423 | 0.21851 | 0.221569 | -0.10249 | 0.00102 | 0.0183 | 0.8183 |
| ***G3*** | -0.05799 | 0.2 382 | 0.11985 | 0.34929 | 0.078561 | 0.07407 | 0.0655 | 0.6334 |
| ***D*** | 0.14729 | 0.6723 | 0.23359 | 0.00431 | -0.23655 | 0.000436 | -0.0235 | 0.2236 |
| ***E*** | 0.14041 | 0.07295 | 0.2366 | 0.119185 | 0.196518 | 0.000365 | 0.09945 | 0.2463 |
| ***P*** | -0.07649 | 0.7543 | -0.09326 | 0.11918 | 0.130432 | 0.16516 | 0.0174 | 0.5291 |
| ***Q*** | -0.04679 | 0.8495 | -0.0661 | 0.00149 | 0.262222 | 0.32561 | -0.01055 | 0.7005 |

**Table 3.** Data on correlation of *GABR* and *MYC* gene expression in group 3 medulloblastoma (where *p* > 0.01 is not significant).

|  | **Group 3α, *MYC*** | | **Group 3β, *MYC*** | | **Group 3γ, *MYC*** | |
| --- | --- | --- | --- | --- | --- | --- |
| **Gene** | **Corr** | **p-value** | **Corr** | **p-value** | **Corr** | **p-value** |
| ***A1*** | -0.4354 | 0.000231 | -0.22611 | 0.178411 | -0.08665 | 0.59496 |
| ***A2*** | 0.37445 | 0.001798 | 0.0169 | 0.920919 | -0.14695 | 0.36556 |
| ***A3*** | 0.16453 | 0.18336 | -0.05419 | 0.750077 | -0.19378 | 0.230879 |
| ***A4*** | 0.09518 | 0.44357 | -0.16961 | 0.315574 | -0.05346 | 0.743189 |
| ***A5*** | 0.329598 | 0.00646 | -0.16255 | 0.336447 | -0.0776 | 0.63410 |
| ***A6*** | -0.29793 | 0.01434 | -0.24294 | 0.147378 | 0.115856 | 0.476525 |
| ***B1*** | -0.3348 | 0.00561 | -0.2602 | 0.11987 | -0.10759 | 0.50873 |
| ***B2*** | -0.05394 | 0.66465 | 0.059709 | 0.725554 | 0.186441 | 0.249361 |
| ***B3*** | 0.310799 | 0.01047 | -0.18055 | 0.2849 | -0.18387 | 0.256068 |
| ***G1*** | -0.17749 | 0.15075 | -0.09118 | 0.591452 | 0.033266 | 0.838527 |
| ***G2*** | -0.32042 | 0.00821 | -0.11099 | 0.513141 | -0.19844 | 0.219629 |
| ***G3*** | 0.28755 | 0.018303 | -0.14109 | 0.404888 | -0.32464 | 0.040973 |
| ***D*** | 0.20138 | 0.10224 | -0.14518 | 0.391261 | -0.13443 | 0.408225 |
| ***E*** | 0.21357 | 0.08269 | 0.084682 | 0.618258 | 0.085304 | 0.60072 |
| ***P*** | 0.34454 | 0.0043 | 0.011098 | 0.948022 | 0.158601 | 0.328332 |
| ***Q*** | 0.31978 | 0.00834 | 0.253068 | 0.130727 | 0.380179 | 0.015527 |

**References**

1. Cavalli FMG, Remke M, Rampasek L, Peacock J, et al (2017) Intertumoral Heterogeneity within Medulloblastoma Subgroups. Cancer Cell 31:737-754. doi: 10.1016/j.ccell.2017.05.005

2. Williams CA, Bell SV, Jenkins A (2010) A residue in loop 9 of the beta2-subunit stabilizes the closed state of the GABAA receptor. J Biol Chem 285:7281-7287. doi: 10.1074/jbc.M109.050294

3. Forkuo GS, Nieman AN, Kodali R, Zahn NM, Li G, Rashid Roni MS, et al (2018) A novel orally available asthma drug candidate that reduces smooth muscle constriction and inflammation by targeting GABAA receptors in the lung. Molec Pharmaceutics 15:1766. Doi: 10.1021/acs.molpharmaceut.7b01013

4. Knutson DE, Kodali R, Divović B, Treven M, Stephen MR, Zahn NM et al (2018) Design and synthesis of novel deuterated ligands functionally selective for the γ-aminobutyric acid type A receptor (GABA_A_R) α6 subtype with improved metabolic stability and enhanced bioavailability. J Medicinal Chem 61:2422. doi: 10.1021/acs.jmedchem.7b01664
